# Plants, fungi, and antifungals: A little less talk, a little more action

**DOI:** 10.1371/journal.ppat.1013395

**Published:** 2025-08-05

**Authors:** George Ly, James M. Bradley, Dario Bonetta, Shelley Lumba

**Affiliations:** 1 Department of Cell and Systems Biology, University of Toronto, Toronto, Ontario, Canada; 2 Ontario Tech University, Oshawa, Ontario, Canada; 3 Centre for the Analysis of Genome Evolution and Function, University of Toronto, Toronto, Ontario, Canada; University of Maryland, Baltimore, UNITED STATES OF AMERICA

## Introduction

Fungal pathogens and infections are understudied relative to other microbial pathogens. This neglect, however, seems unwarranted as fungi negatively impact both food security and human health, causing crop losses of up to 40% worldwide and close to four million deaths annually [1]. Today, fungal diseases exceed malaria or breast cancer cases and rival the number of tuberculosis and HIV infections annually [1]. As new fungal pathogens emerge due to climate change, these numbers will only grow, increasing our need for antifungal agents [2]. Typically, only four classes of antifungal drugs are used to treat human fungal infections, which primarily target the fungal cell wall or ergosterol in cell membranes [3]. With the limited biochemical range of antifungals, resistance is arising in both farmers’ fields and hospitals, and occasionally these resistances cross both clinical and agricultural boundaries [4]. The urgent threat to human health and growing resistance to the limited number of antifungal agents, led the World Health Organization to make an urgent call for research into the world’s most dangerous fungi [5]. To address these challenges, how do we find new classes of antifungals and identify new druggable targets in fungi?

### 1. Rooting for antifungals

Discovering lead compounds usually involves some sort of screening, whether through rational methods like molecular modeling, biochemistry, and AI or by phenotypic screening of compounds against cells. Historically, however, some of our most successful antibiotics were found not by high-throughput screening but by noting ecological interactions between organisms. *Penicillium* mold makes penicillin to inhibit bacteria, and streptomycin exists for *Streptomyces* species to gain an advantage over other microorganisms in the soil [6]. With respect to fungal interactions with other organisms, one of their major partners are plants and in particular, plant roots in the soil.

Plants successfully colonized land by forming a complex web of mutualistic interactions with fungi [7]. As they were swept onto shores, ancestral land plants had limited means to obtain nutrients from soil due to a lack of root systems. Fossil evidence from 400 million years ago revealed mycorrhizal fungi growing inside plant cells, pointing to an ancient relationship where fungi served as a root system [8]. These associations are now essential to the health and diversity of both kingdoms in natural ecosystems and in agriculture [7,9]. Whether beneficial or pathogenic, these inter-kingdom courtships must involve dialogue, and small molecules appear to be the words that both plants and fungi understand [10].

**Fig 1:**
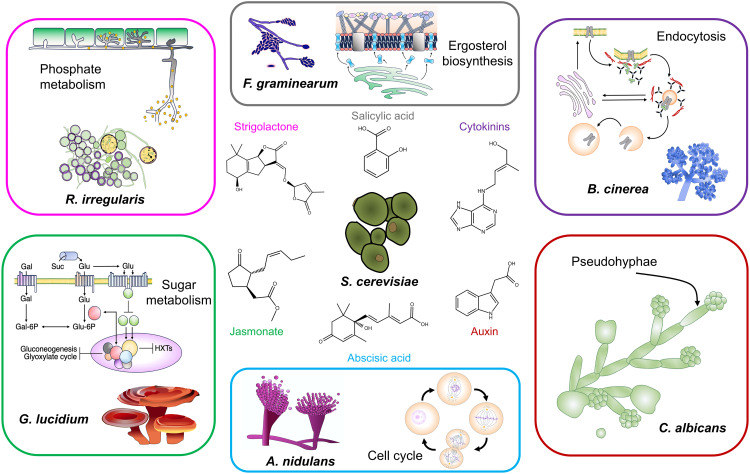
Fungi that respond to plant hormones. Examples of fungi and processes that are annotated as responsive to the plant hormones: abscisic acid, auxin, cytokinins, jasmonate, salicylic acid, strigolactone. Each process is labelled in the box containing a fungal species (in bold). *Saccharomyces cerevisiae* (center) can be used as a model system to study the processes each of the plant hormones target in fungi. Abscisic acid regulates transcription of cell-cycle genes in *Aspergillus nidulans* [13]. Auxin induces pseudohyphal growth in both *Candida albicans* and *S. cerevisiae* [14]. Cytokinins inhibit the growth of *Botrytis cinerea* by misregulating endocytosis [17]. Jasmonate treatment decreases accumulation of proteins involved in sugar metabolism in *Ganoderma* [18]. Salicylic acid disrupts the cell membrane integrity of *Fusarium graminearum* by inhibiting ergosterol biosynthesis [25]. Strigolactone triggers a change in phosphate metabolism of *Rhizophagus irregularis* leading to symbiosis [48].

Plants, for example, make eight small molecule hormones that are exuded into the soil, and six of them are known to influence fungal processes (**Fig 1**) [11–20]. Many fungi respond to and in some cases, produce these plant small molecules. While it is not surprising that plant-associated fungi like *Botrytis cinerea* produce auxin presumably to manipulate its plant partner, the human fungal pathogen, *Candida albicans*, can also make and respond to auxin [21–24]. The ability of auxin to stimulate filamentation in *C. albicans* suggests that it has an important endogenous signaling role in fungi that do not associate with plants [24]. Beyond auxin, salicylic acid can both inhibit the growth of *Fusarium graminearum* under acidic conditions and serve as a carbon source for *Fusarium* under basic conditions [25]. *F. graminearum*, *Ustilago maydis*, *Aspergillus niger*, and *Epichloë festucae* express a salicylate hydroxylase which allows the breakdown and use of salicylic acid as a nutrient source [26–29]. Although the reasons why fungi synthesize plant hormones are not entirely clear, responses to plant small molecules are plentiful across the fungal kingdom.

One of the most studied classes of plant symbiotic small molecule signals in fungi are strigolactones (SLs), a collection of related compounds that attract arbuscular mycorrhizal (AM) hyphae to the plant root [16]. AM fungi associate with over 80% of plant species and upon colonizing a root, the fungi give up nutrients, particularly inorganic phosphate (Pi), in exchange for plant carbon [9]. Although the SL example is the most prevalent in the literature, there are other examples of plant hormones eliciting fungal responses, which means that fungal receptors and signaling pathways must exist for these plant small molecules [12]. Decoding these chemical signals in fungi will unlock new classes of compounds that exploit fungal processes and identify proteins in fungi that bind these small molecules.

### 2. The yeast of my worries

Since some plant-associated fungi like AM fungi can be genetically intractable, it is challenging to dissect the mechanisms by which fungi respond to small molecules [30]. Genetic tools in fungi such as *Fusarium graminearum, Botrytis cinerea,* and *Ustilago maydis*, however, have elucidated mechanisms of plant-fungal communication, particularly in complex ecological interactions [31–34]. For example, mutants in *Ustilago* demonstrate that the ability of *Ustilago* to acquire organic acids from its maize host is critical to the virulence of the pathogen [35]. To accelerate the translation of the molecular dialogue between plants and fungi, we need a fungal model with a well-annotated genome on which we can conduct high-throughput genetic and genome-wide screens. *Saccharomyces cerevisiae* (yeast), is well positioned to serve as a fungal model due to the breadth of genetic and genomic tools, along with the depth of understanding of its genetics and its historically close association with plant substances [36].

Yeast is fully sequenced and annotated. It has complete gene knockout and overexpression collections. But perhaps yeast’s most useful attribute is its ability to grow under defined conditions, so physiological and biochemical experiments are easily designed. Although lab-friendly by contrast to other fungi, yeast is poorly adapted to natural environments like soil, thus it may have lost many responses to plant-derived compounds [37]. Nevertheless, yeast does have conserved pathways relevant to physiological responses and developmental programs that are typically experienced in the wild. For example, although non-pathogenic, mixing yeast with mammalian macrophages identified glyoxylate cycle activation as important in pathogenic fungal responses to phagocytosis [38]. The synthetic ability of yeast to interact and exchange nutrients with algae indicates that yeast can interact with photosynthetic organisms [39]. Perhaps most importantly, yeast has many features found in other fungi, such as polarized growth, adhesion, and biofilm formation [40]. For yeast to serve as a useful model of plant-fungal communication, yeast must also remember how to respond to plant small molecules. Transcriptomic approaches can reveal which molecules yeast remembers and which ones it has forgotten during domestication.

### 3. Words matter

Querying the potential of yeast to respond to plant hormones or other small molecules requires a robust, broad, and unbiased phenotyping tool. In this context, transcriptomics fits well. Indeed, treating yeast with SLs and methyl jasmonate results in distinct gene expression profiles, suggesting that specific processes are modulated by these hormones [41]. In the case of SL, yeast responds by inducing genes involved in the phosphate starvation response [41]. Using a combination of yeast genetics and AI-based structural analysis, the high-affinity phosphate transporter, Pho84, was identified as an SL sensor.

A plant molecule, therefore, binds and inhibits the function of a yeast protein, but is this relationship conserved in “real-world fungi”? First, many fungal species, including medically important ones, have a binding pocket in Pho84, where SL appears to bind [41]. Second, in whole-organism assays, SL modulates phosphate homeostasis in the pathogenic fungus, *Fusarium graminearum*, and the endophytic symbiont, *Serendipita indica* [41]. Finally, SLs are crucial for plants to establish associations with AM fungi under Pi starvation. Although AM fungi are distantly related to yeast, we do not think it is a coincidence that SL specifically modulates phosphate homeostasis in diverse fungal species.

### 4. Spreading the word

The identification of Pho84 as a target of SL, has implications in the development of antifungal lead compounds. In *Cryptococcus neoformans*, for example, a knockout line that ablated *PHO84* and related homologs, disrupted fungal growth in phosphate-limited conditions within the host and reduced the ability of the pathogen to form an extracellular capsule normally required for full virulence [42]. Moreover, *PHO84* affects the ability of *Candida albicans* to cause disease and survive attacks by host macrophages and neutrophils [43]. Interestingly, loss of *PHO84* function changes levels of cell wall polysaccharides in *C. albicans*. Since other antifungals target cell walls, targeting Pho84 with SL-like molecules may enhance the antifungal activity of these molecules [43]. Crucially, humans do not encode *PHO84* homologs, and phosphate metabolism in humans is fundamentally different from fungi, making fungal *PHO84* homologs promising drug targets [44]. Finally, the SL binding pocket in yeast Pho84 shares conserved features across many fungal species, suggesting Pho84 homologs may also be druggable [41]. Indeed, amino acid substitutions in the SL binding site of Pho84 in yeast alter the responsiveness of yeast to certain fungicides, indicating that these are druggable sites [45].

### 5. What does it all mean?

Plants and fungi interact in a two-way street, with both partners sending signals to affect processes in the other. Phosphate metabolism appears to be a major target for both partners. The plant pathogenic fungi, *Magnaporthe* and *Colletotrichum*, mis-regulate phosphate metabolism in the plant through protein effectors, while plants use the small molecule hormone, SL to alter phosphate metabolism in a variety of fungi [41,46]. When plants are low in phosphate, plants produce and exude more SLs in soil [47]. As an endogenous plant hormone, SL sculpts shoot and root architecture to optimize growth and obtain more phosphate in the soil. Along the same vein, emitted SLs promote associations with fungi to provide additional phosphate. Intriguingly, plant-derived SL impacts the very same metabolic pathway of the nutrient, phosphate, that it needs to acquire from the fungus. The meaning of the SL signal in both plant and fungal kingdoms appears to be phosphate. Is this startling similarity simply a coincidence, or does it suggest an intriguing origin of plant small molecule hormones?

Perhaps plant hormones functioned initially as ecological signals to coordinate ancestral plant–fungal symbioses, which later became endogenous signals to regulate development, as plants evolved more complex structures. The first land plants required fungi to process minerals and anchor plants to rocks. In this sense, fungi were the first plant roots, and therefore, plants needed to control them. To do this, perhaps plants mimicked core fungal compounds, which could explain why non-plant-associated fungi like *C. albicans* make and respond to auxin [12]. Consistent with this, non-vascular plants like liverworts, which do not have plant SL receptors, make SL-like compounds that stimulate AM fungal associations [20]. Whatever the case, plants could not evolve signals to communicate with fungi without fungi already having the ability to listen. If true, how many compounds do fungi make that plants have learnt to mimic? Similarly, are there plant compounds that fungi have coopted to manipulate plants? These plant symbiosis signals represent an untapped resource of natural compounds that target precise processes in fungi, with wide-ranging implications to address the growing threat of human and agricultural fungal infections.
